# Differential transcriptional regulation of the *NANOG* gene in chicken primordial germ cells and embryonic stem cells

**DOI:** 10.1186/s40104-021-00563-5

**Published:** 2021-03-04

**Authors:** Hee Jung Choi, So Dam Jin, Deivendran Rengaraj, Jin Hwa Kim, Bertrand Pain, Jae Yong Han

**Affiliations:** 1 Department of Agricultural Biotechnology and Research Institute of Agriculture and Life Sciences, Seoul National University, Seoul, 08826 South Korea; 2 Univ Lyon, Universite ´Lyon 1, INSERM, INRAE, Stem Cell and Brain Research Institute, U1208, USC1361, 69500 Bron, France; 3 Institute for Biomedical Sciences, Shinshu University, Minamiminowa, Nagano, 399-4598 Japan

**Keywords:** Chicken, Embryonic stem cells, *NANOG* gene, Primordial germ cells, Regulatory elements

## Abstract

**Background:**

*NANOG* is a core transcription factor (TF) in embryonic stem cells (ESCs) and primordial germ cells (PGCs). Regulation of the *NANOG* gene by TFs, epigenetic factors, and autoregulatory factors is well characterized in ESCs, and transcriptional regulation of *NANOG* is well established in these cells. Although *NANOG* plays a key role in germ cells, the molecular mechanism underlying its transcriptional regulation in PGCs has not been studied. Therefore, we investigated the mechanism that regulates transcription of the chicken *NANOG* (*cNANOG)* gene in PGCs and ESCs.

**Results:**

We first identified the transcription start site of *cNANOG* by 5′-rapid amplification of cDNA ends PCR analysis. Then, we measured the promoter activity of various 5′ flanking regions of *cNANOG* in chicken PGCs and ESCs using the luciferase reporter assay. *cNANOG* expression required transcriptional regulatory elements, which were positively regulated by *POU5F3* (*OCT4)* and *SOX2* and negatively regulated by *TP53* in PGCs. The proximal region of the *cNANOG* promoter contains a positive transcriptional regulatory element (CCAAT/enhancer-binding protein (*CEBP*)-binding site) in ESCs. Furthermore, small interfering RNA-mediated knockdown demonstrated that *POU5F3*, *SOX2*, and *CEBP* played a role in cell type-specific transcription of *cNANOG*.

**Conclusions:**

We show for the first time that different *trans*-regulatory elements control transcription of *cNANOG* in a cell type-specific manner. This finding might help to elucidate the mechanism that regulates *cNANOG* expression in PGCs and ESCs.

## Background

Gene transcription is mainly regulated by transcription factors (TFs) that bind to specific DNA sequences (called motifs) located in the promoter regions of genes [1]. Many TFs contribute to tissue- and cell type-specific gene transcription according to their recognition specificity [2–4]. In addition, TFs generally initiate and guide cell fate such as lineage progression and control the stability of cell differentiation [5]. Therefore, identification of regulatory elements within the promoter region is considered crucial to understand the mechanism underlying transcriptional regulation in specific cell types. A germ cell-specific gene regulatory network is required to maintain the unique properties of primordial germ cells (PGCs) for transmission of genetic information to the next generation [6]. Many studies have investigated germ cell-specific gene promoters to understand their regulatory mechanisms. In many species, germ cells have a unique mechanism of transcription initiation that uses alternate forms of core promoter elements [7–10]. Also, germ cells reorganize different type of core promoter TFs under the control of germ cell-specific TFs during germ cell differentiation [11–13].

In mammals, core TFs such as *NANOG, OCT4,* and *SOX2* control maintenance of pluripotency. Core TFs play an important role in establishing control of gene expression programs that define the identity of embryonic stem cells (ESCs) [14–16]. In particular, the *NANOG* gene is important for acquisition of pluripotency by ESCs and embryonic germ cells (EGCs) [17–19]. Several earlier studies identified the regulatory elements of *NANOG* that are required to maintain the self-renewal and pluripotency of ESCs [20–22]. The major regulators of *NANOG* expression are Octamer- and Sox-binding elements present at the upstream of transcription start site (TSS) in its promoter region, and these elements are positively regulated by binding of *OCT4* and *SOX2* in ESCs [20, 23]. Direct binding of *ZFP143* to the proximal region of the *NANOG* promoter regulates *NANOG* expression by modulating *OCT4* binding [24]. In addition, TF-binding *cis*-regulatory elements of *NANOG*, including *SP1/SP3-*, *SALL4-*, and *BRD4*-binding sites, have been identified as positive regulators [25–27]. On the other hand, *P53-*binding sites negatively regulate *NANOG* expression to induce differentiation of ESCs [28]. Therefore, regulation of *NANOG* expression plays a critical role in determining the fate of pluripotent cells.

PGCs express several pluripotency-related TFs such as *NANOG, POU5F3*, and *SOX2*, and their expression controls transcription of germness-related genes in these cells [11, 29]. *NANOG* plays an essential role during early germ cell development as a key TF required for the formation of PGCs and maintenance of early germ cells [30, 31]. *NANOG*-deficient PGCs reportedly undergo apoptotic death [32]. It was recently reported that *NANOG* regulates PGC-specific epigenetic programming and global histone methylation [33, 34]. *NANOG* is evolutionarily conserved in mammals and most of the lower vertebrate species, including chicken. In particular, *NANOG* orthologs from chicken, zebrafish, and axolotl are highly conserved [35–37]. Similar to mammals, *NANOG* is crucial to maintain pluripotency and self-renewal of chicken ESCs [35]. *NANOG* is expressed during chicken intrauterine embryonic development and is exclusively expressed in PGCs from Hamburger and Hamilton stage 5 (HH5) to HH8. Therefore, *NANOG* is also important to maintain pluripotency and cell proliferation in chicken intrauterine embryos and PGCs [31, 35, 38].

Despite the exclusive expression of *NANOG* in chicken PGCs, the molecular mechanism that regulates its transcription in these cells has not been fully clarified. This study investigated enhancers and suppressors of the proximal promoter region of the chicken *NANOG* (*cNANOG*) gene in PGCs and ESCs. Furthermore, we investigated transcriptional control of *cNANOG* expression via *trans*-regulatory elements and TFs, which are important for its cell type-specific expression.

## Methods

### Experimental design, animals, and animal care

This study investigated the *cis-* and *trans-*regulatory elements that are important for modulating transcription of the *NANOG* gene in chicken PGCs using the dual luciferase assay and transcriptome analysis. The management of White Leghorn (WL) chickens was approved by the Institute of Laboratory Animal Resources, Seoul National University, Korea (SNU-190401-1-1). The chickens were housed according to standard procedures at the University Animal Farm, Seoul National University, Korea.

### 5′ Rapid amplification of cDNA ends (5′-RACE) PCR analysis

To determine the TSS of the *cNANOG* gene (Gene ID: 100272166), 5′-RACE PCR was performed using a GeneRacer Kit (Invitrogen, Carlsbad, CA, USA) following the manufacturer’s instructions. Gene Racer RNA Oligo-ligated mRNA was reverse-transcribed into cDNA. Single-stranded cDNA served as the template in nested 5′-RACE PCR using the GeneRacer 5′ Primer and reverse gene-specific primers (GSPs). The *cNANOG* reverse GSP was 5′-GTC TGC AGT AGG GCT AGT GGC AGA GTC T-3′. The RACE products were identified by DNA sequencing analysis. To confirm the quality of adapter-ligated RNA, 5′-RACE PCR was performed with a chicken β-actin reverse GSP, which was 872 bp in size and contained 828 bp of β-actin and 44 bp of the GeneRacer RNA Oligo.

### Construction of NanoLuc luciferase expression vectors derived from the *cNANOG* promoter

To construct NanoLuc luciferase expression vectors, the 5′ flanking region of the *cNANOG* gene was amplified using genomic DNA extracted from adult chicken blood and inserted into the pGEM-T Easy vector (Promega, Madison, WI, USA). Primer sets were used to clone differently sized fragments of the *cNANOG* promoter (Table 1). Then, different lengths of the 5′ upstream region of the *cNANOG* gene were inserted between the *KpnI* and *XhoI* sites of the pNL1.2 vector (Promega).

**Table 1:** List of primer sequences used to clone the *NANOG* promoter

Primer name	Primer sequence (5′ → 3′)
*cNANOG* − 3550 bp_F	AAGCTTTGTCCTTTTCTTGACC
*cNANOG* − 3375 bp_F	CTGGAGTCAAGGGCTGTGG
*cNANOG* − 3154 bp_F	TGGGCCCCTCGTTACAGCT
*cNANOG* − 2928 bp_F	CCAGCAGTACAAGCTCCGAA
*cNANOG* − 1988 bp_F	GCGACACGTGGAACA
*cNANOG* − 945 bp_F	CATGGGGGTGTCTGCTC
*cNANOG* − 627 bp_F	CTTCTTTGTGCTCCTCC
*cNANOG* − 442 bp_F	CTGCAGTCTGCAATGC
*cNANOG* − 407 bp_F	AATGTCCCGGGGGGGTCTCTGG
*cNANOG* − 377 bp_F	CCATTCTTTGTACTTGGGTGGGGACCGATGAG
*cNANOG* − 312 bp_F	CGAGGGCGGGGGTGCCAGCCCAG
*cNANOG* − 250 bp_F	CTGCAGTCTGCTCCTCC
*cNANOG* − 210 bp_F	CTGCAGTCTGCAATGC
*cNANOG* − 170 bp_F	CCAAAGGGGGAAGCTGC
*cNANOG* − 130 bp_F	ACTCTCCGAATATCCCCATAGC
*cNANOG* − 69 bp_F	TCGTGACAATCTCTTG
*cNANOG* promoter_R	GGTCGGGACGACACCT

### Luciferase reporter assay

The Nano-Glo Dual Reporter Assay System (Promega) was used to assess *cNANOG* promoter activity. Prepared cells were seeded in a 96-well plate and co-transfected with the pGL4.53 firefly luciferase (Fluc) and pNL1.2 (NlucP/cNANOG RE) NanoLuc luciferase (Nluc) plasmids using Lipofectamine 2000 (Invitrogen). After transfection for 24 h, cells were lysed with lysis buffer containing Fluc substrate. Fluc signals were then quenched, followed by reaction with Nluc substrate. Signals in arbitrary units (AU) of Nluc and Fluc were measured using a luminometer (Glomax-Multi-Detection System; Promega). Promoter activities were calculated by determining the ratio of Nluc/Fluc signals in AU. pNL1.2, an empty vector, was used as a negative control. All reporter assays were repeated at least three times.

### Culture of chicken PGCs, ESCs, and DF-1 cells

WL PGCs were maintained and sub-passaged in KnockOut DMEM (Thermo Fisher-Invitrogen, USA) supplemented with 20% fetal bovine serum (Hyclone, South Logan, UT, USA), 2% chicken serum (MilliporeSigma, Burlington, MA, USA), 1× nucleosides (MilliporeSigma), 2 mmol/L *L*-glutamine, 1× nonessential amino acids, β-mercaptoethanol, 10 mmol/L sodium pyruvate, 1× antibiotic-antimycotic (ABAM; Thermo Fisher-Invitrogen), and 10 ng/mL human basic fibroblast growth factor (MilliporeSigma). PGCs were sub-cultured onto mitomycin-inactivated mouse embryonic fibroblasts at an interval of 5–6 d via gentle pipetting.

Chicken ESCs were generously provided by Dr. Bertrand Pain (INSERM-INRAE). These cells were maintained and sub-passaged as previously described [39]. Briefly, ESCs were cultured in 50 mL of DMEM/F12 (GIBCO, Grand Island, NY, USA) supplemented with 10% fetal bovine serum (Hyclone), 1× nonessential amino acids, 10 mmol/L sodium pyruvate, β-mercaptoethanol, 1× ABAM (Thermo Fisher-Invitrogen), 5 ng/mL insulin-like growth factor 1, 1 ng/mL stem cell factor, 1 ng/mL interleukin 6, 1 ng/mL soluble interleukin 6 receptor α, and 1000 U/mL human leukemia inhibitory factor. ESCs were sub-cultured onto mitotically inactivated STO cells.

Chicken DF-1 cells (CRL-12203; American Type Culture Collection, USA) and chicken embryonic fibroblasts (CEFs) were cultured as negative controls. Chicken DF-1 cells were maintained and sub-passaged in DMEM (Hyclone) supplemented with 10% fetal bovine serum (Hyclone) and 1× ABAM (Thermo Fisher-Invitrogen). CEFs were derived from 6-day-old WL embryos and maintained in DMEM (Hyclone) supplemented with 10% fetal bovine serum (Hyclone) and 1× ABAM (Thermo Fisher-Invitrogen). All chicken cells (PGCs, ESCs, DF-1 cells, and CEFs) were cultured in an incubator at 37 °C under an atmosphere of 5% CO_2_ and 60–70% relative humidity.

### Prediction of putative TF-binding elements

TF-binding sites were predicted by MatInspector, a Genomatix program (http://www.genomatix.de/) using TRANSFAC matrices (vertebrate matrix; core similarity 1.0 and matrix similarity 0.8), and PROMO 3.0, which uses TRANSFAC version 8.3 (http://alggen.lsi.upc.es/cgi-bin/promo_v3/promo/promoinit.cgi?dirDB=TF_8.3).

### Small interfering RNA (siRNA)-mediated knockdown of predicted TFs

siRNAs targeting predicted TFs were designed using siRNA Target Finder (http://www.ambion.com) (Table 2). Commercially available control siRNA (sense: 5′-CCU ACG CCA CCA AUU UCG U-3′) was purchased from Bioneer Corporation (Daejeon, Korea). To validate the knockdown efficiency of predicted TFs, PGCs or ESCs were transfected with 50 pmol of siRNAs targeting CCAAT/enhancer-binding protein (*CEBP)* genes, including *CEBPA, CEBPB, CEBPD, CEBPG,* and *CEBPZ,* and *TP53* using Lipofectamine 2000 (Invitrogen). After siRNA transfection for 24 h, the knockdown efficiencies of the predicted TFs and the effects on *cNANOG* gene transcription were measured by quantitative reverse-transcription PCR (RT-qPCR).

**Table 2:** List of siRNA sequences targeting each transcription factor for knockdown analysis

Target gene	siRNA sequence (5′ → 3′)	
	Sense	Antisense
*POU5F3*	UGGCUCAAUGAGGCAGAGA	UCUCUGCCUCAUUGAGCCA
*SOX2*	AACCAAGACCCUGAUGAAG	CUUCAUCAGGGUCUUGGUU
*TP53*	UCAUGGACCUCUGGAGCAU	AUGCUCCAGAGGUCCAUGA
*CEBPA*	GCGAGGAGGAGGAGGUGA	UUCACCUCCUCCUCCUCGC
*CEBPB*	GCGCAAGAGCCGCGACAAA	UUUGUCGCGGCUCUUGCGC
*CEBPD*	ACGAGAAGCUGCACAAGAA	UUCUUGUGCAGCUUCUCGU
*CEBPG*	AAAUUAAGCUCCUGACCAA	UUGGUCAGGAGCUUAAUUU
*CEBPZ*	GAGAAAAGCAAGAAGGAAA	UUUCCUUCUUGCUUUUCUC

### Analysis of gene expression by RT-qPCR

Total RNA was extracted from test samples using TRIzol reagent (Molecular Research Center, USA) in accordance with the manufacturer’s protocol and reverse-transcribed using the Superscript III First-Strand Synthesis System (Invitrogen). The PCR mixture contained 2 μL of PCR buffer, 1 μL of 20× EvaGreen qPCR dye (Biotium, Hayward, CA, USA), 0.4 μL of 10 mmol/L dNTP mixture, and 10 pmol each of gene-specific forward and reverse primers (Table 3). RT-qPCR was performed in triplicate. Relative target gene expression was quantified after normalization against chicken glyceraldehyde 3-phosphate dehydrogenase (*GAPDH*) expression as an endogenous control.

**Table 3:** List of primer sequences used for quantitative real-time PCR

Gene symbol	Primer sequence (5′ → 3′)	
	Forward	Reverse
*CEBPA*	CCCACCTGCAGTACCAGATC	TCTTTTTGGATTTGCCGCGG
*CEBPB*	CGCCCGCCTTTAAATCCATG	GGGCTGAAGTCAATGGCTCT
*CEBPD*	ACTTCTACGACGCCAAGGTG	CTCTCGTCCTCGTACATGGC
*CEBPG*	CCCACAGCTAACGTGTCAGT	GGACGGGCTCTTCTTTGACA
*CEBPZ*	CGCTGTTCACAGTCTCCACT	GGACGCTGTGAGAAAGACCA
*SOX2*	AAACCGAGCTGAAACCTCCC	TGTGCATCTTCGGGTTCTCC
*SOX3*	CGGCTCAGCAGACTCGATAC	TCGCCGTGGCTTAAGAACTT
*POU5F3*	TGAAGGGAACGCTGGAGAGC	ATGTCACTGGGATGGGCAGAC
*TP53*	CCGTGGCCGTCTATAAGAAA	ACAGCACCGTGGTACAGTCA
*NANOG*	AGTGGCAGAGTCTGGGGTAT	ACTACTACTGGCCCTCTCCG
*GAPDH*	GGTGGTGCTAAGCGTGTTAT	ACCTCTGTCATCTCTCCACA

### Statistical analysis

Statistical analysis was performed using GraphPad Prism (GraphPad Software, La Jolla, CA, USA). Significant differences between groups were determined by a one-way analysis of variance with Bonferroni’s multiple comparison test and the unpaired t-test. A value of *P* < 0.05 indicated statistical significance.

## Results

### Identification of the TSS of the *cNANOG* gene

**Fig. 1:**
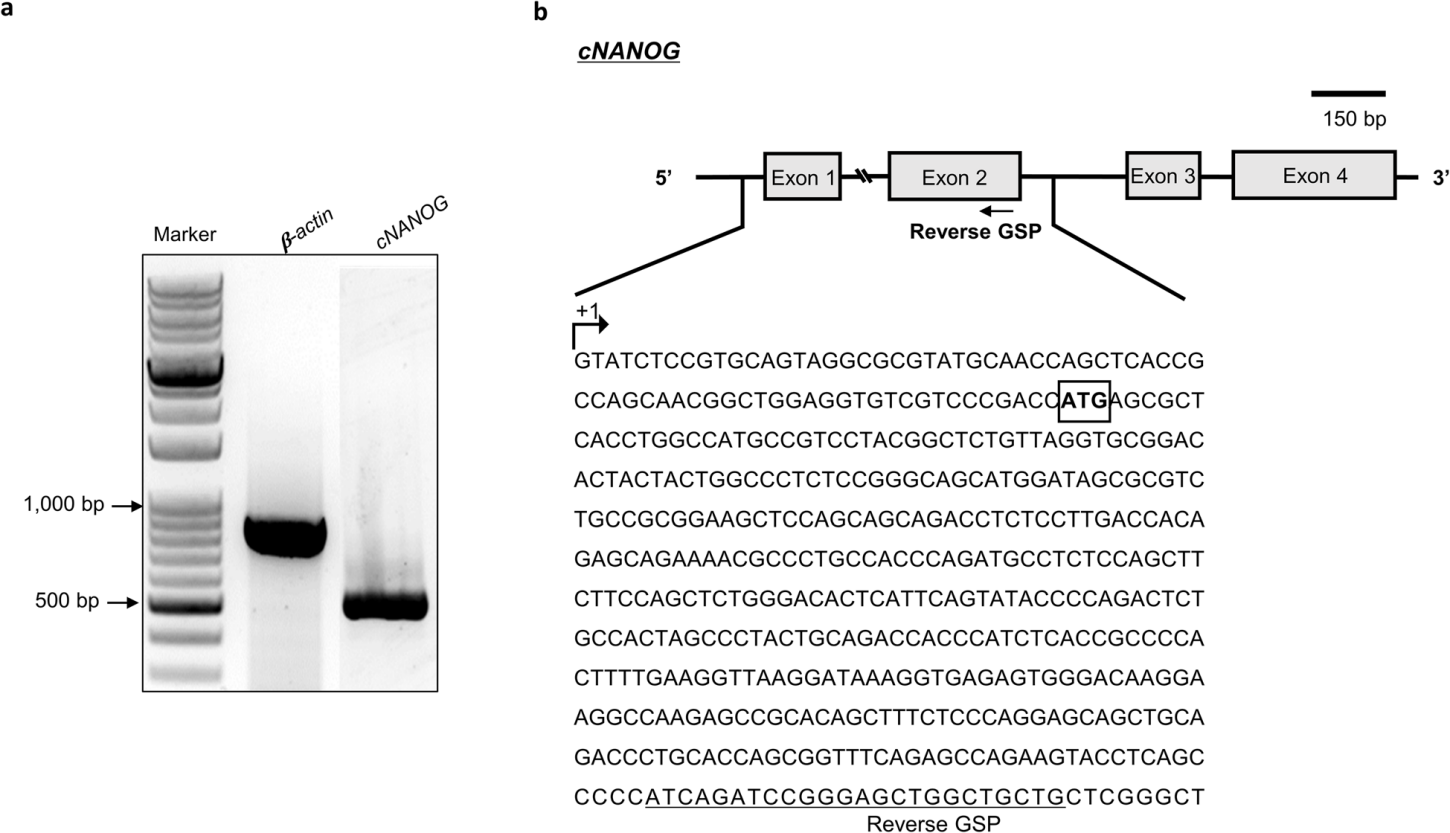
Identification of the transcription start site (TSS) of the chicken *NANOG* (*cNANOG*) gene by 5′-rapid amplification of cDNA ends (RACE) analysis. **a** After performing 5′-RACE, the PCR product was analyzed and its size was determined by agarose gel electrophoresis. Scale bar = 150 bp. **b** The 5′-RACE product was cloned into the pGEM-T vector and sequenced. The TSS of the *cNANOG* gene is located 70 bp upstream of the translation start codon ATG. + 1 indicates the potential TSS of the *cNANOG* gene

To better understand transcriptional regulation of the *cNANOG* gene, we first determined the TSS of this gene by 5′-RACE PCR analysis. A 470 bp PCR product was obtained using a reverse GSP that targeted exon 2 of the *cNANOG* gene (Fig. 1a and b). Sequencing analysis identified the TSS of the *cNANOG* gene located 70 bp upstream of the ATG start codon (Fig. 1b).

### Characterization of the *cNANOG* core promoter in PGCs and ESCs

**Fig. 2:**
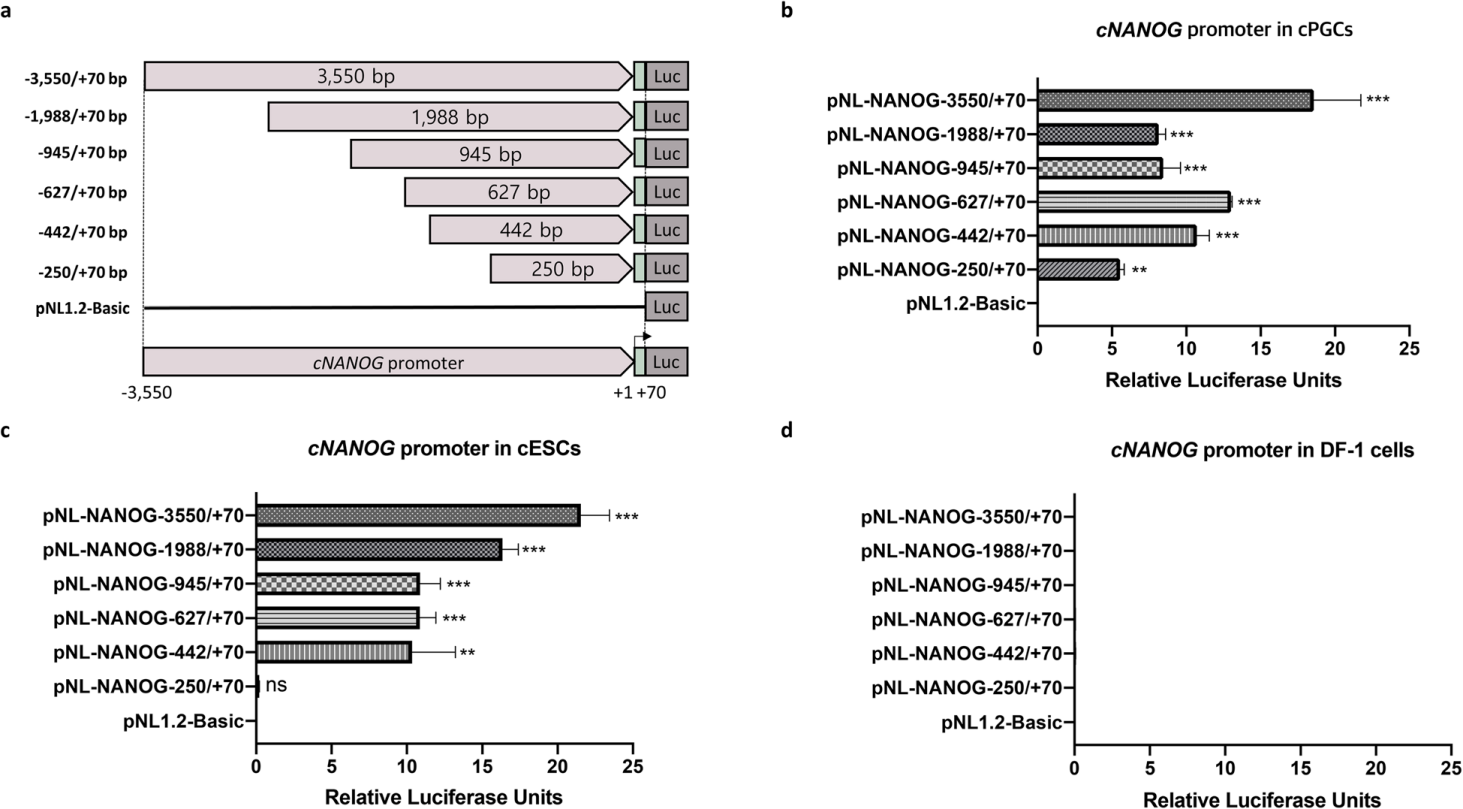
Promoter variants reduce activity of the chicken *NANOG* (*cNANOG*) gene in a cell type-dependent manner. **a** Schematic diagram of deletion of the *cNANOG* gene promoter (− 3550/+ 70 bp). Relative luciferase activity in chicken primordial germ cells (PGCs) **(b)**, chicken embryonic stem cells (ESCs) **(c)**, and DF-1 cells **(d)**. Luciferase activity was normalized against firefly luciferase expression (pNL1.2-Basic) to control for variation in the transfection efficiency. Significant differences are indicated as ns (no significance), ** *P* < 0.01, and *** *P* < 0.001. Error bar represent the SEs for three replicate reactions

To investigate the proximal region of the core promoter of the *cNANOG* gene, we generated a series of 5′ deletion luciferase reporter constructs of the 6− region, which were randomly designed based on the − 3550/+ 70 bp sequence (Fig. 2a). Luciferase activity derived from differently sized fragments of the *cNANOG* promoter was examined in PGCs, ESCs, and DF-1 cells transfected with the constructs for 24 h using Lipofectamine 2000. Luciferase activity was 4-fold higher in PGCs transfected with the − 3550/+ 70 bp fragment than in PGCs transfected with the − 250/+ 70 bp fragment (Fig. 2b). On the other hand, the − 250/+ 70 bp fragment did not exhibit luciferase activity in ESCs (Fig. 2c). None of the *cNANOG* promoter fragments were active in DF-1 cells (Fig. 2d). These results suggest that transactivation level of the complete promoter (− 3550/+ 70 bp sequence) was similar between PGCs and ESCs but *cNANOG* transcription was differentially regulated in PGCs and ESCs by the proximal enhancer.

### *POU5F3* and *SOX2* regulate constitutive expression of *cNANOG* in PGCs

**Fig. 3:**
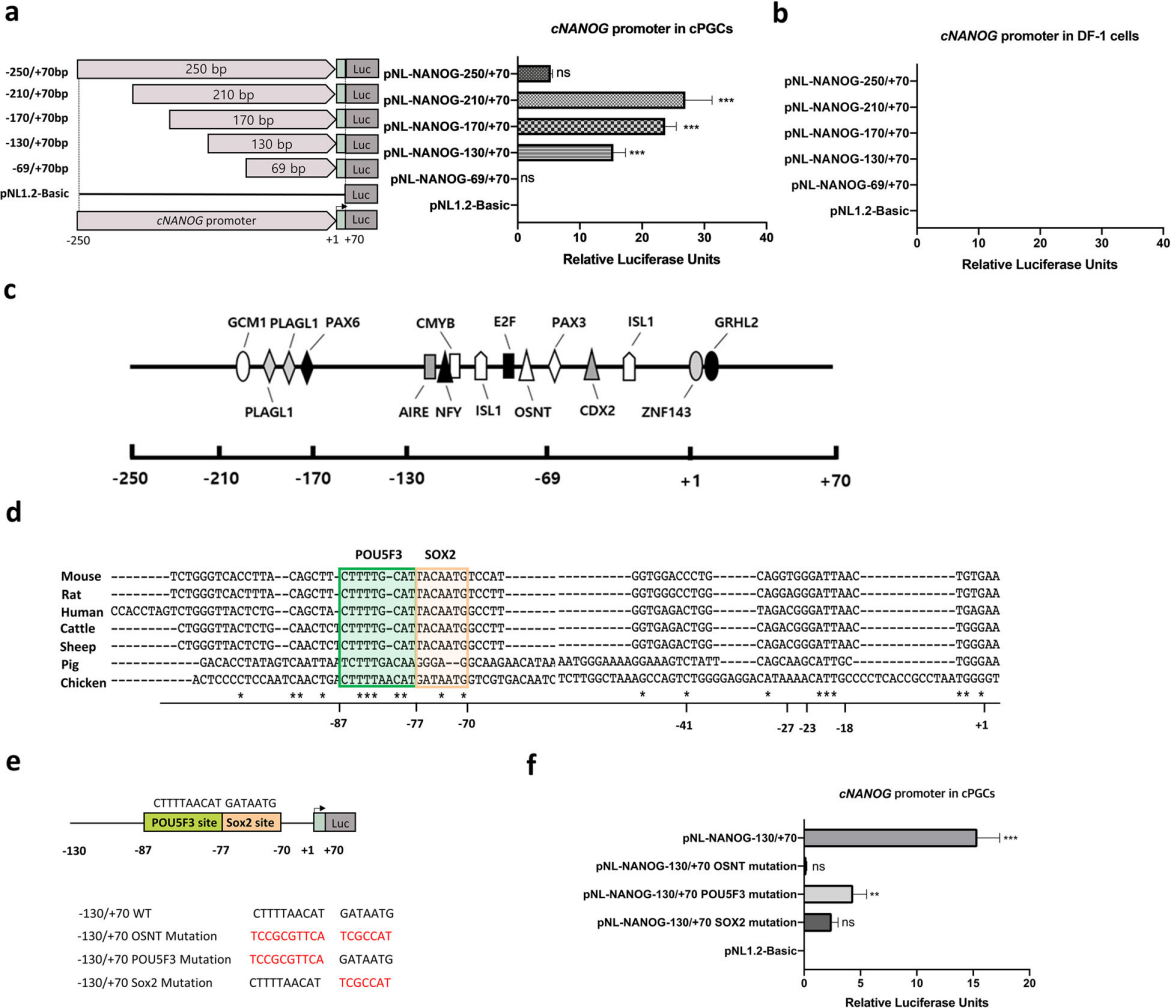
Verification of the proximal enhancer of the chicken *NANOG* (c*NANOG* gene) in chicken primordial germ cells (PGCs). **a-b** Schematic diagram of the constructed *cNANOG* promoter vectors and luciferase activity in PGCs **(a)** and DF-1 cells **(b)**. **c** Prediction of transcription factor (TF)-binding sites in the *cNANOG* promoter region located from − 250 to + 70 bp. **d** Multiple alignment of the putative *cNANOG* proximal enhancer with transcriptional regulatory elements of *NANOG* genes from mouse, rat, human, cattle, sheep, pig, and chicken. Prediction of mostly conserved *POU5F3-* and *SOX2-*binding sites in chicken. **e** Mutation analysis of putative *POU5F3-* and *SOX2-*binding sites in PGCs. **f** Luciferase activity of the −130/+ 70 bp *cNANOG* promoter fragment compared with that of mutated promoter constructs. Significant differences are indicated as ** *P* < 0.01 and *** *P* < 0.001. Error bar represent the SEs for five replicate reactions

To further examine PGC-specific *cNANOG* promoter activity and binding to the proximal enhancer, we generated four constructs harboring fragments of the − 250/+ 70 bp region of the *cNANOG* promoter via deletion of the 5′ upstream region. Among the four constructs, the − 210/+ 70 bp, − 170/+ 70 bp, and − 130/+ 70 bp fragments still showed promoter activity in PGCs, while the − 69/+ 70 bp fragment did not (Fig. 3a). None of the *cNANOG* promoter fragments were active in DF-1 cells (Fig. 3b). These results suggest that a positive transcriptional regulatory element is located between − 130 and − 69 bp in PGCs.

Based on the findings regarding *cNANOG* promoter activity described above, we predicted TFs with binding sites located between − 130 and − 69 bp of the *cNANOG* promoter using two software programs (PROMO and MatInspector). Several TF-binding sites, including *AIRE-, NFY-, CMYB-, ISL1-, E2F-,* and *OSNT* (*OCT4/* POU5F3*, SOX2, NANOG*, and *TCF3*)-binding sites, were identified in this region (Fig. 3c). Sequence alignment of this *cNANOG* promoter region from six vertebrate species showed that the *POU5F3*- and *SOX2*-binding regulatory elements are highly conserved in mammalian species (Fig. 3d). To determine the functional contributions of the *POU5F3-* and *SOX2*-binding sites to constitutive expression of *cNANOG*, site-directed mutagenesis, which can disturb the recruitment of TFs, was performed (Fig. 3e). Mutation of the *POU5F3/SOX2*-binding sites in the 200 bp fragment (− 130/+ 70 bp) significantly reduced relative luciferase activity in PGCs. Moreover, relative luciferase activity was reduced significantly more by mutation of the *SOX2*-binding site alone than by mutation of the *POU5F3*-binding site alone in PGCs (Fig. 3f). Taken together, these results suggest that *POU5F3* and *SOX2* play a role in transcription of *cNANOG* by directly binding to the 5′ upstream promoter region in PGCs.

### *TP53* suppresses *cNANOG* gene expression in PGCs

**Fig. 4:**
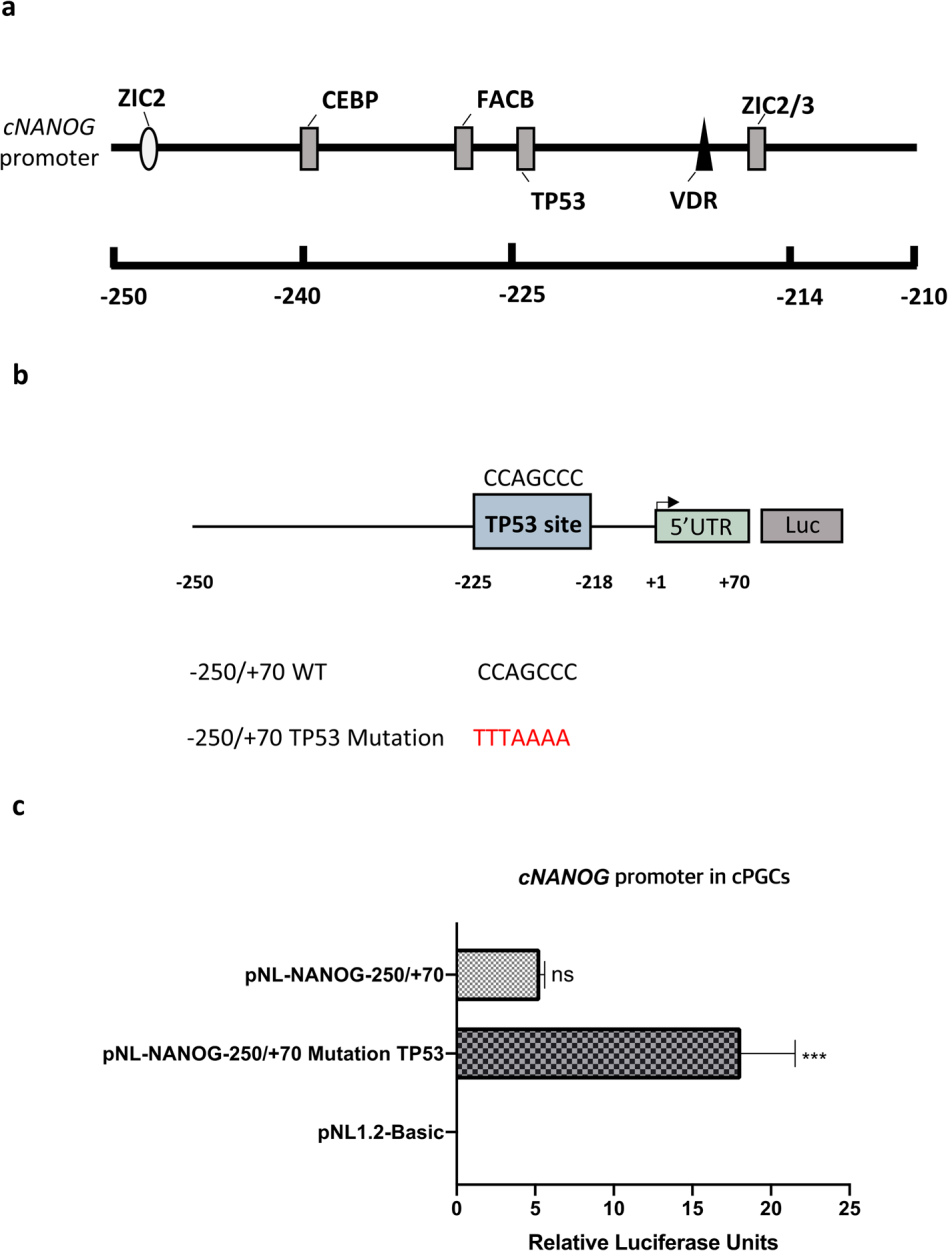
Negative regulation of chicken *NANOG* (c*NANOG*) gene expression by *TP53* in chicken primordial germ cells (PGCs). **a** Prediction of transcription factor (TF)-binding sites in the c*NANOG* promoter region from − 250 to −210 bp. **b** Mutation analysis of putative *TP53*-binding sites in PGCs. **c** Luciferase activity of pNL-NANOG − 250/+ 70 and *TP53*-binding site-mutated (pNL-NANOG − 250/+ 70 TP53 mutation) vectors. pNL1.2-Basic was used as a control. Significant differences are indicated as ns (no significance) and *** *P* < 0.001. Error bar represent the SEs for five replicate reactions

Luciferase activity was at least 3-fold higher in PGCs transfected with the − 210/+ 70 bp, − 170/+ 70 bp, and − 130/+ 70 bp fragments than in PGCs transfected with the − 250/+ 70 bp fragment (Fig. 3a). These results suggest that a negative transcriptional regulatory element is located between − 250 and − 210 bp. To investigate the suppression of *cNANOG* promoter activity, we predicted TFs that have binding sites within this region using two software programs (PROMO and MatInspector) (Fig. 4a). Among the predicted TFs, *TP53* is a suppressor of *NANOG* transcription, while *ZIC2/3* and *CEBP* are positive regulators of *NANOG* transcription [28, 40, 41]. We further examined whether *TP53* affects *cNANOG* promoter activity in PGCs by performing site-directed mutagenesis and comparing the mutant with the wild-type − 250/+ 70 bp fragment (Fig. 4b). Deletion of the *TP53-*binding site in the *cNANOG* promoter region significantly increased luciferase activity in PGCs (Fig. 4c). These results demonstrate that *TP53* suppresses *cNANOG* transcription in PGCs.

### *CEBP* transactivates the *cNANOG* promoter in ESCs

**Fig. 5:**
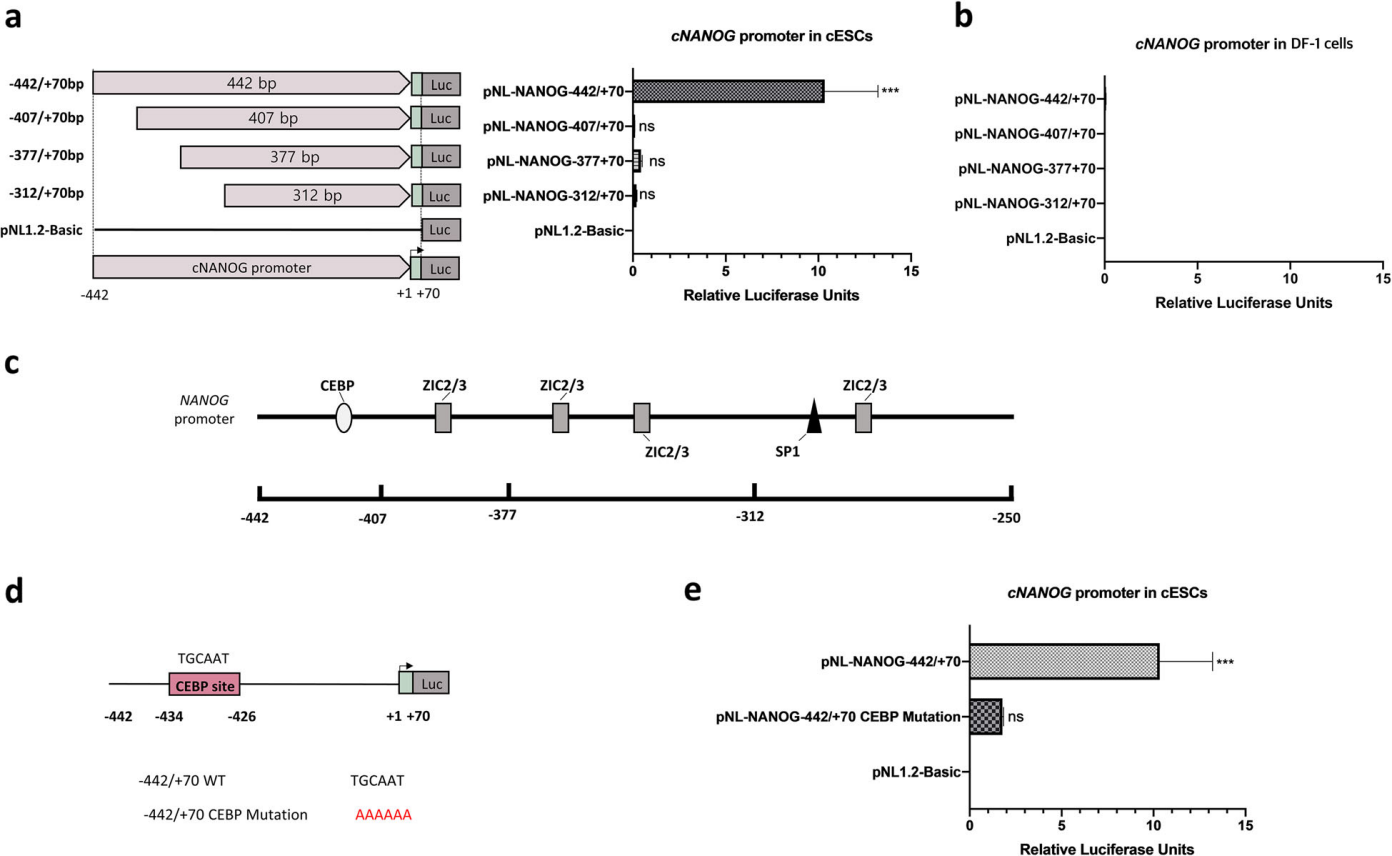
Verification of the proximal enhancer of the chicken *NANOG* (*cNANOG*) gene in chicken embryonic stem cells (ESCs). **a-b** Schematic diagram of the constructed *cNANOG* promoter vectors and luciferase activity in ESCs **(a)** and DF-1 cells **(b)**. **c** Prediction of transcription factor (TF)-binding sites in the *cNANOG* promoter region from − 442 to − 250 bp. **d** Mutation analysis of putative CCAAT/enhancer-binding protein (*CEBP*)-binding sites in ESCs. **e** Luciferase activity of the − 442/+ 70 bp *cNANOG* promoter fragment compared with that of the mutated promoter. Significant differences are indicated as ns (no significance) and *** *P* < 0.001. Error bar represent the SEs for five replicate reactions

To further investigate the potential transcriptional regulatory elements in ESCs, we generated four constructs harboring fragments of the − 442/+ 70 bp region of the *cNANOG* promoter via deletion of the 5′ upstream region. Among the four constructs, the − 407/+ 70 bp, − 377/+ 70 bp, and − 312/+ 70 bp fragments exhibited significantly reduced *cNANOG* promoter activity in ESCs (Fig. 5a). None of the *cNANOG* promoter fragments were active in DF-1 cells (Fig. 5b). These results suggest that a positive transcriptional regulatory element is located between − 442 and − 407 bp in ESCs.

We analyzed the − 442/+ 70 bp fragment using two software programs (PROMO and MatInspector) to identify important TF-binding sites that maintain the basal activity of the *cNANOG* gene in ESCs. Only a *CEBP*-binding site was identified between − 442 and − 407 bp (Fig. 5c). To examine the effect of the *CEBP-*binding site on promoter activity, we constructed vectors containing mutations of this site in the − 422/+ 70 bp fragment (Fig. 5d). Mutation of the *CEBP*-binding site in the − 442/+ 70 bp region dramatically reduced relative luciferase activity in ESCs compared with the wild-type construct of the same region (Fig. 5e). Taken together, these results suggest that *CEBP* positively regulates transcription of *cNANOG* by directly binding to the 5′ upstream promoter region in ESCs.

### Effects of predicted TFs on *cNANOG* gene transcription

**Fig. 6:**
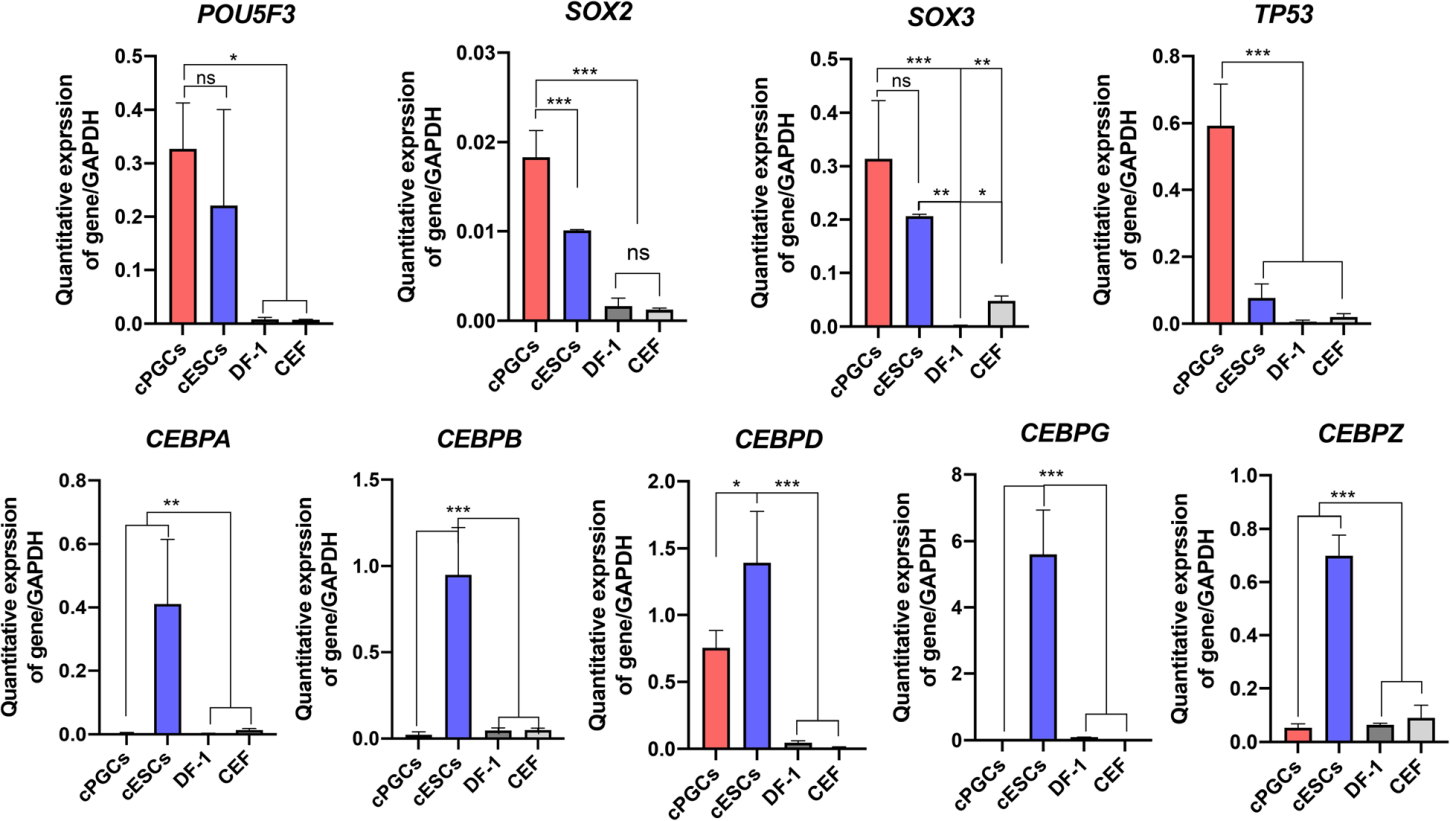
Quantitative expression analysis of predicted transcription factors (TFs) in various cell types. Expression of predicted TFs in chicken primordial germ cells (PGCs), embryonic stem cells (ESCs), DF-1 cells, and chicken embryonic fibroblasts (CEFs) was analyzed by quantitative reverse-transcription PCR (RT-qPCR). Error bars indicate the standard deviation of triplicate analyses. Significant differences are indicated as ns (no significance), * *P* < 0.05, ** *P* < 0.01, and *** *P* < 0.001

To confirm that the predicted TFs are expressed in PGCs and ESCs, we conducted RT-qPCR using RNA prepared from PGCs, ESCs, DF-1 cells, and CEFs. Expression of chicken *CEBP* genes (*CEBPA, CEBPB, CEBPD, CEBPG*, and *CEBPZ*) was significantly higher in ESCs than in other cells. By contrast, expression of *POU5F3* and *SOX2/3* was significantly higher in PGCs and ESCs than in DF-1 cells and CEFs. Expression of *POU5F3* and *SOX3* did not differ between PGCs and ESCs, while *SOX2* was significantly upregulated in PGCs. Additionally, expression of *TP53* was significantly higher in PGCs than in other cells (Fig. 6).

**Fig. 7:**
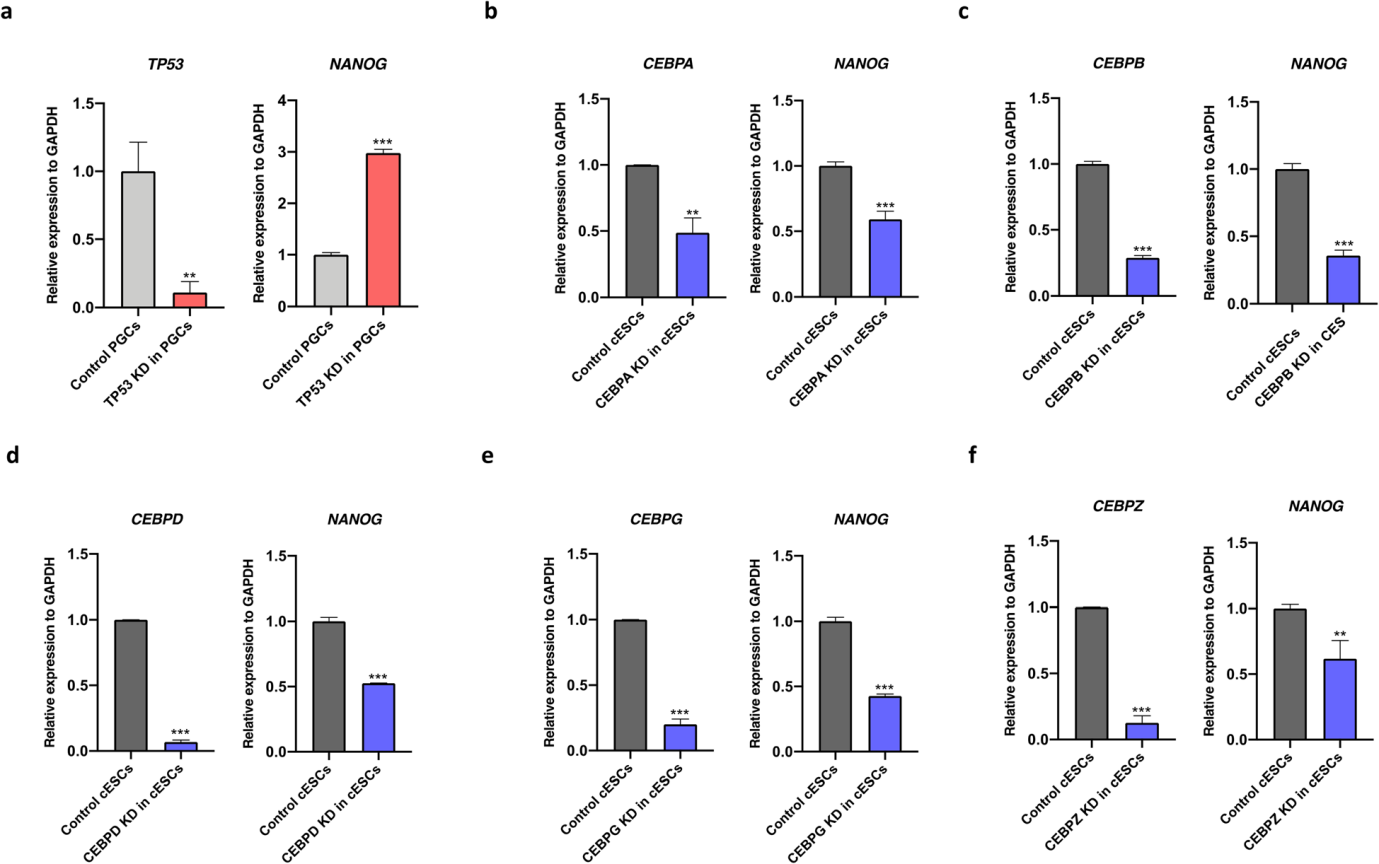
Relative gene expression analysis after knockdown of predicted transcription factors (TFs) in cultured primordial germ cells (PGCs) and embryonic stem cells (ESCs). **a** Efficiency of small interfering RNA (siRNA)-mediated knockdown of *TP53* in PGCs was analyzed by quantitative reverse-transcription PCR (RT-qPCR). Relative expression of *NANOG* was determined. **b–f** Efficiency of siRNA-mediated knockdown of *CEBPA*, *CEBPB*, *CEBPD*, *CEBPG*, and *CEBPZ* in ESCs was analyzed by RT-qPCR. Relative expression of *NANOG* was determined in each sample. Error bars indicate the standard deviation of triplicate analyses. Significant differences are indicated as * *P* < 0.05, ** *P* < 0.01, and *** *P* < 0.001

**Fig. 8:**
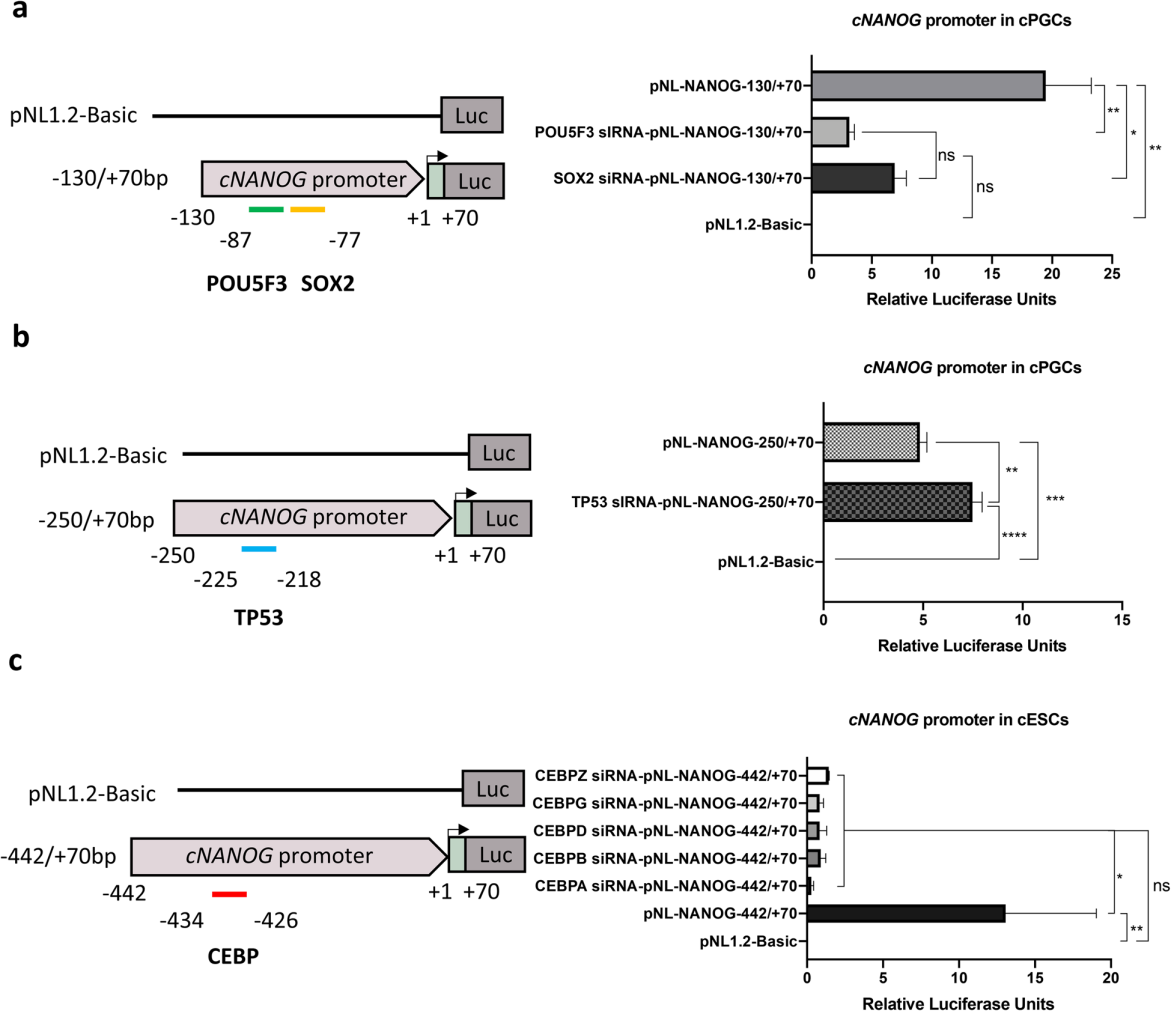
Chicken *NANOG* promoter activity after knockdown of predicted transcription factors. **a** Luciferase activity of pNL-NANOG-130/+ 70 after the knockdown of *POU5F3* and *SOX2* in chicken primordial germ cells (PGCs). **b** Luciferase activity of pNL-NANOG − 250/+ 70 after the knockdown of *TP53* in chicken PGCs. **c** Luciferase activity of pNL-NANOG − 442/+ 70 after the knockdown of *CEBPA, CEBPB, CEBPD, CEBPG*, and *CEBPZ* in chicken embryonic stem cells (ESCs). Error bars indicate the standard deviation of triplicate analyses. Significant differences are indicated as ns, no significance, * *P* < 0.05, ** *P* < 0.01, *** *P* < 0.001, and **** *P* < 0.0001

We further examined whether these TFs affect the transcription of *cNANOG* in PGCs and ESCs using a siRNA-mediated knockdown assay. Knockdown of *TP53* significantly increased *cNANOG* expression in PGCs, indicating that *TP53* decreases *cNANOG* transcription (Fig. 7a). Knockdown of *CEBPA*, *CEBPB*, *CEBPD*, *CEBPG,* and *CEBPZ* significantly decreased *cNANOG* gene expression in ESCs (Fig. 7b–f). We also examined the luciferase activities driven by c*NANOG* promoter containing wild type binding sites after the knockdown of predicted TFs in PGCs and ESCs (Fig. 8). Knockdown of *POU5F3* and *SOX2* significantly reduced the activity of the c*NANOG* promoter fragment (− 130/+ 70 bp) containing wild type binding sites, whereas, knockdown of *TP53* is significantly increased the activity of the c*NANOG* promoter − 250/+ 70 bp fragment in PGCs (Fig. 8a and b). Knockdown of *CEBPA*, *CEBPB*, *CEBPD*, *CEBPG,* and *CEBPZ* in ESCs dramatically reduced the activity of the c*NANOG* promoter − 442/+ 70 bp fragment containing wild type *CEBP* binding site (Fig. 8c). These results indicate that these TFs control transcription of *cNANOG* by directly interacting with its promoter in a cell type-specific manner.

## Discussion

The homeodomain TF *NANOG* is important to maintain pluripotency in mammalian pluripotent cells during embryonic development [17]. Therefore, many studies have been conducted to determine how NANOG expression is regulated by core factors in mammalian stem cells [20, 22, 23]. In addition, its expression is required for the formation of germ cells [30] and maintained in proliferating PGCs during the migration [42]. It has been recently reported that regulatory elements of NANOG transcription in PGCs are different from the ES cells in mice but key regulatory factors have not yet been identified [43]. In chicken, *NANOG* was also important for maintaining the pluripotency in PGCs and ESCs [31, 35, 38, 44]. However, the molecular mechanisms that regulate transcription of the *NANOG* gene in chicken PGCs and ESCs remain unclear. In this regard, we characterized the structure of *cNANOG* and analyzed its promoter activity in chicken PGCs and ESCs.

We successfully transcribed *cNANOG* under the control of the proximal regulatory region located within 130 bp upstream of the TSS in PGCs. Furthermore, we identified the regulatory region of *cNANOG* located within 442 bp upstream of the TSS in ESCs. Moreover, we showed that *TP53* suppresses *cNANOG* transcription in PGCs. These results suggest that the *cNANOG* promoter functions in a cell type-specific manner. Similarly, Yeom et al. reported that the mouse *Oct4* gene contains two separate regulatory elements [45]. The distal regulatory element is specifically active in mouse ESCs and EGCs, while the proximal enhancer is active in the epiblast. Thus, transcription of the mouse *Oct4* gene is regulated in a stage-specific manner. Our findings indicate which elements are critical for gene expression in PGCs. This is the first report of a transcriptional regulatory factors of *NANOG* that is differentially active in a cell type-specific manner in chicken.

Many researchers have studied mammalian ESCs to determine which core factors regulate the *NANOG* gene. Most of the positive regulation of *NANOG* transcription has been discovered in the proximal region, which encompasses *OCT3/4* and *SOX2* in mouse ESCs. This region is strongly conserved in various mammalian species [20, 23]. Mutation of Octamer- and Sox-binding sites dramatically reduces transcription of *NANOG.* Therefore, *OCT3/4* and *SOX2* play an important role in regulation of the *NANOG* gene promoter in mammalian ESCs [23]. Also, these TFs such as *POU5F3, SOX2/3, KLF2, *and* SALL4* are highly expressed in chicken ES cells and PGCs [46]. According to the comparison of genomic sequence elements, core pluripotency factors of the mouse are not conserved with chicken [47]. In the present study, mutation of *POU5F3*- and *SOX2*-binding sites in the proximal region significantly reduced c*NANOG* promoter activity in PGCs. Although the DNA sequences of *POU5F3* and *SOX2,* which are recognized by mouse core pluripotency factors, are not well conserved in chicken, *POU5F3* and *SOX2* are key regulators of c*NANOG* transcription. Further investigation by the electrophoretic mobility shift assay and chromatin immunoprecipitation sequencing is required to determine the core TFs in chicken PGCs.

Programmed death of PGCs is essential to remove abnormal, misplaced, and excess cells during PGC development and this is important to establish the next generation. In *Drosophila melanogaster*, *TP53* is reportedly involved in elimination of excess PGCs during PGC development [48] and, mouse PGCs are regulated by p53 to process the PGCs apoptosis [49]. In addition, *TP53* binds to the *NANOG* promoter and suppresses *NANOG* expression for maintenance of genome stability in ESCs [28]. Interestingly, our results showed that the *TP53-*binding site negatively controlled *NANOG* transcription in chicken PGCs. Therefore, we propose that *TP53* plays important roles in the regulation of *NANOG* transcription to maintain genome stability in PGCs.

*CEBPB* interacts with *p300* to modulate histone acetylation [50], and *p300* is a co-activator that binds to *NANOG* for maintenance of pluripotency in ESCs [51]. In our study, *CEBPA, CEBPB, CEBPD, CEBPG,* and *CEBPZ* were significantly upregulated in chicken ESCs. In addition, knockdown of these TFs dramatically decreased transcription of *cNANOG* in chicken ESCs. These results suggest that *CEBP* in chicken ESCs participate in regulation of *cNANOG* transcription by directly interacting with putative binding sites in the *cNANOG* promoter.

As described above, transcription regulation of c*NANOG* is conserved in mammals, although DNA sequences of regulation factors differ between chicken and mammals. Typically, mammalian PGCs can be induced by cell signaling [52]. Interestingly, mouse *Nanog* is key regulator of PGCs-like cells independent of *BMP4* and *Wnt* signals by activating the expression of germ cell-specific TFs [33]. On the other hand, chicken germ cells may be specified by maternally inherited factors like *VASA* and *DAZL* in germ plasm [53, 54]. Recently, the epigenetic regulation of *NANOG* in chicken PGCs has been investigated by our group to understand the molecular mechanisms involved in the specification of germ cells [34]. However, the regulation of c*NANOG* in chicken germ cell specification is still unclear. In this study, we shown that chicken *NANOG* has differential regulatory roles in PGCs and ESCs, even though c*NANOG* promoter region sharing the common transcription factor binding sites. These finding provided insights into germ cell and stem cell-specific transcriptional regulatory mechanisms.

## Conclusion

**Fig. 9:**
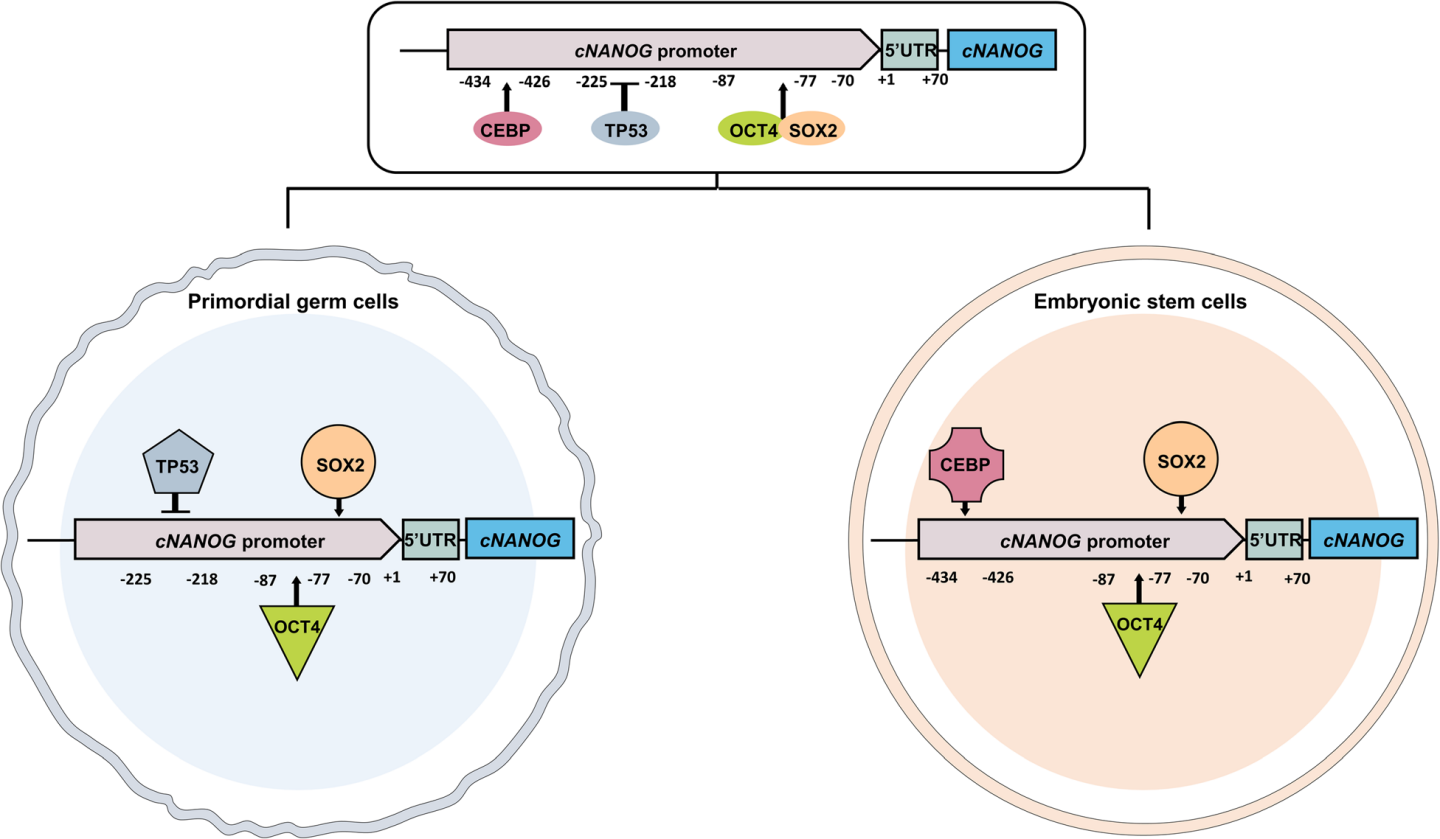
A model illustrating regulation of chicken *NANOG* (*cNANOG*) gene transcription in chicken primordial germ cells (PGCs) and embryonic stem cells (ESCs). *cNANOG* gene expression requires transcriptional *trans*-regulatory elements that are positively controlled by *POU5F3* and *SOX2* and negatively controlled by *TP53* in PGCs. On the other hand, CCAAT/enhancer-binding protein (*CEBP*) positively regulates *cNANOG* gene expression in ESCs

This study demonstrated that the proximal regulatory region of the *cNANOG* gene differs between PGCs and ESCs. We showed that the *cNANOG* gene is positively regulated by *POU5F3* and *SOX2* and negatively regulated by *TP53* in PGCs, while it is positively regulated by *CEBP* in ESCs. Collectively, these findings aid understanding of transcriptional regulation of the *cNANOG* gene in PGCs and ESCs (Fig. 9).

## Data Availability

The datasets during and/or analyzed during the current study available from the corresponding authors on reasonable request.

## Abbreviations

TF: Transcription factor
ESCs: Embryonic stem cells
PGCs: Primordial germ cells
cNANOG: Chicken NANOG
CEBP: CCAAT/enhancer-binding protein
EGCs: Embryonic germ cells
TSS: Transcription start site
HH5: Hamburger and Hamilton stage 5
WL: White Leghorn
5′-RACE: 5′ Rapid amplification of cDNA ends
GSPs: Gene-specific primers
Fluc: Firefly luciferase
Nluc: NanoLuc luciferase
AU: Arbitrary units
CEF: Chicken embryonic fibroblasts
GAPDH: Glyceraldehyde 3-phosphate dehydrogenase
